# Diagnostic and predictive molecular biomarkers in brain tumors across the lifespan: an age-stratified consensus statement

**DOI:** 10.1007/s11060-025-05329-x

**Published:** 2025-11-24

**Authors:** Angela Mastronuzzi, Enrico Franceschi, Federica D’Antonio, Elisa Bennicelli, Giulia Berzero, Eugenia Cella, Massimo Filippi, Gaetano Lanzetta, Enrico Marchioni, Claudia Milanaccio, Matteo Simonelli, Paola Bini, Antonio Silvani, Andrea Pace

**Affiliations:** 1 Department of Hematology/Oncology, Cell and Gene Therapy, Scientific Institute for Research, Hospitalization and Healthcare (IRCCS), Bambino Gesù Children’s Hospital, Rome, Italy; 2 Nervous System Medical Oncology Department, IRCCS Istituto delle Scienze Neurologiche di Bologna / AUSL di Bologna, Via Altura 3, 40139 Bologna, Italy; 3 Department of Experimental Medicine, Sapienza University of Rome, Rome, Italy; 4 Medical Oncology Unit 2, IRCCS Ospedale Policlinico San Martino, 16132 Genoa, Italy; 5 Neurology Unit, IRCCS San Raffaele Hospital, Milan, Italy; 6 Faculty of Medicine, Vita-Salute San Raffaele University, Milan, Italy; 7 Department of Internal Medicine and Medical Specialties (DiMI), School of Medicine, University of Genova, Genova, Italy; 8 Department of Oncology, San Paolo Hospital, Savona, Italy; 9 Neuroimaging Research Unit, Division of Neuroscience, IRCCS San Raffaele Scientific Institute, Vita-Salute San Raffaele University, Milan, Italy; 10 Department of Medical Oncology and Palliative Care, Casa di Cura INI, Grottaferrata, Italy; 11 Neuro-Oncology and Neuroinflammation Unit, IRCCS Mondino Foundation, Pavia, Italy; 12 Neuro-Oncology Unit, Department of Pediatric Hematology and Oncology, IRCCS Istituto Giannina Gaslini, Genova, Italy; 13 Department of Medical Oncology and Hematology, IRCCS Humanitas Research Hospital, Rozzano, Italy; 14 Department of Biomedical Sciences, Humanitas University, Pieve Emanuele, Italy; 15 Neuro-Oncology Unit, Fondazione IRCCS Istituto Neurologico Carlo Besta, Milan, Italy; 16 Neuro-Oncology Unit, IRCCS Regina Elena National Cancer Institute, Rome, Italy

**Keywords:** Predictive and prognostic biomarkers, Brain tumors, Precision medicine, Age-stratified profiling, Targeted therapy

## Abstract

**Background:**

Molecular profiling has significantly advanced neuro-oncology, enabling the integration of biomarkers into the diagnosis and management of brain tumors. Precision medicine is emerging as a promising strategy; however, the marked heterogeneity of central nervous system tumors results in a low prevalence of actionable targets, limiting clinical applicability. Despite these challenges, ongoing progress in genetics and molecular biology offers new opportunities for targeted therapies. The incidence and clinical relevance of biomarkers vary across tumor types and age groups, reflecting the biological complexity of brain neoplasms throughout life.

**Methods:**

A multidisciplinary expert panel conducted a systematic review of the literature and developed a consensus statement addressing key predictive biomarkers across pediatric, adolescent and young adult (AYA), adult, and elderly populations. Evidence was evaluated for diagnostic, prognostic, and therapeutic relevance.

**Results:**

Clinical benefit from targeted therapies has been demonstrated for a limited number of alterations, including **BRAF p.V600E**, **NTRK fusions**, **EGFR**, **H3 K27M**, and** IDH1/2 mutations**, while several additional biomarkers remain under investigation. The consensus provides an age-stratified overview of these molecular alterations and discusses challenges such as variability in testing approaches, interpretation of variants of uncertain significance, and limited access to comprehensive molecular diagnostics.

**Conclusion:**

Based on current evidence and expert opinion, the statement highlights the need for age-adapted testing strategies, multidisciplinary molecular tumor boards, and increased clinical trial availability for patients with rare or emerging biomarkers. These recommendations aim to support the implementation of precision medicine and improve outcomes across all age groups.

## Introduction

The management of brain tumors has entered a transformative era with the advent of molecular diagnostics and precision medicine. The 2021 World Health Organization (WHO) Classification of Tumors of the Central Nervous System (CNS) and the most recent guidelines from the European Association for Neuro-Oncology (EANO) both underscore the central importance of integrating molecular biomarkers into routine clinical practice for diagnosis, prognosis, and therapeutic decision-making [1, 2]. This paradigm shift has led to the systematic incorporation of molecular and clinical data, enabling the identification and validation of novel biomarkers with diagnostic, prognostic, and predictive relevance [3].

The clinical impact of molecular diagnostics is increasingly evident. The use of biomarkers such as IDH mutations, MGMT promoter methylation, and BRAF alterations has already improved risk stratification, informed therapeutic choices, and, in select contexts, contributed to better survival and reduced treatment-related toxicity, particularly in pediatric and young adult populations [4, 5]. For example, the identification of actionable gene fusions in pediatric gliomas has enabled the use of targeted therapies that offer durable responses with fewer side effects compared to conventional regimens.

Despite these advances, the prevalence of actionable molecular alterations in brain tumors remains low – less than 5% in most adult and elderly cohorts –constraining the broad clinical application of targeted therapies and highlighting the ongoing need for robust translational research and clinical trials [1]. In contrast, certain pediatric brain tumors are characterized by a higher incidence of actionable gene fusions and MAPK pathway alterations, further emphasizing the profound biological heterogeneity of CNS neoplasms across the lifespan [4, 6].

Age is now recognized as a key biological determinant of the molecular landscape in brain tumors. Pediatric, adolescent and young adult (AYA), adult, and elderly patients each exhibit distinct molecular profiles, with important implications for tumor classification, risk stratification, and therapeutic responsiveness [7]. For example, pediatric gliomas are enriched for MAPK pathway alterations and gene fusions, while adult gliomas more commonly harbor IDH mutations, TERT promoter mutations, and EGFR amplifications [8]. The AYA population represents a unique transitional group with overlapping molecular features, and the elderly population is characterized by a predominance of IDH-wildtype glioblastomas and a distinct spectrum of actionable alterations [7, 9].

The clinical utility of predictive biomarkers also varies by age group and tumor subtype. While some biomarkers, such as IDH mutations, have established roles in guiding therapy, others remain investigational or are relevant only within specific molecular and clinical contexts [10, 11]. The interpretation of variants of uncertain significance (VUS), the harmonization of testing protocols, and the integration of molecular data into multidisciplinary decision-making –often via molecular tumor boards –are ongoing challenges in the implementation of precision medicine in neuro-oncology [12].

Given these complexities, there is a pressing need for consensus-based recommendations that address the incidence, clinical significance, and practical application of predictive molecular biomarkers in brain tumors, stratified by age. This consensus statement, developed by a multidisciplinary panel of experts, provides a comprehensive overview of current evidence on predictive biomarkers in brain tumors across the lifespan. We review the most relevant biomarkers by tumor type and age group, discuss their prognostic, and therapeutic implications, and offer practical recommendations for clinical practice and future research. Our aim is to support clinicians, researchers, and policymakers in the optimal use of molecular biomarkers to advance personalized care and improve outcomes for patients with brain tumors of all ages.

## Methods

This consensus statement was developed by a multidisciplinary panel of experts representing key specialties, including pediatric oncology, neuro-oncology, neurology, neuropathology, molecular biology, genetics, and clinical research. Panelists were selected based on their recognized expertise, publication record, and active involvement in clinical and research networks relevant to brain tumor management.

**Fig. 1:**
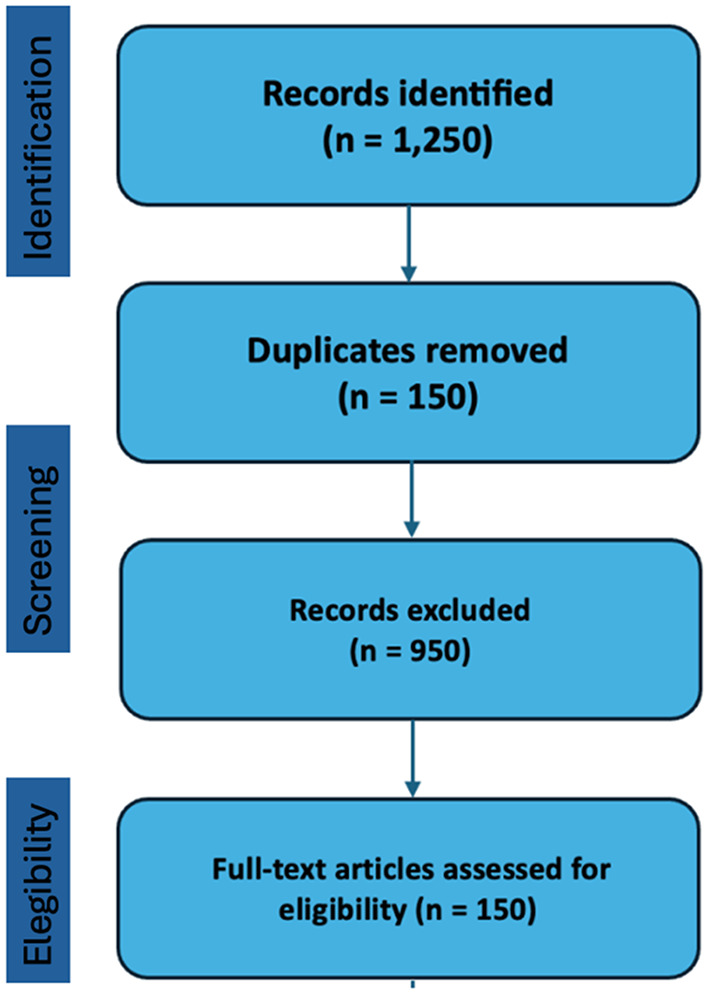
Flow diagram summarizing the consensus development process

A comprehensive and systematic literature review was conducted to identify relevant studies published up to March 2025. The databases searched included PubMed, Embase, Scopus, and Web of Science. Search terms combined “brain tumor,” “glioma,” “medulloblastoma,” “ependymoma,” “meningioma,” “biomarker,” “molecular profiling,” “precision medicine,” “targeted therapy,” “pediatric,” “AYA,” “adult,” “elderly,” and “age stratification.” Additional sources included the most recent international guidelines (e.g., WHO CNS 2021, EANO), consensus statements, and major clinical trials. Reference lists of key articles were also screened for additional relevant studies (Fig. 1).

### Consensus process

To ensure transparency and clarity, the consensus process will be described in a fully discursive manner. The project adopted a structured, modified Delphi methodology, involving fourteen panelists with expertise in oncology, neurology, and internal medicine. Each panelist was allocated to a subgroup, and these subgroups were responsible for systematically reviewing the literature on predictive biomarkers, focusing on well-defined tumor types and age groups such as pediatric, adolescent and young adult, adult, and elderly populations. For each assigned area, subgroup members drafted sections that summarized the incidence and clinical relevance of specific biomarkers, while also highlighting practical limitations observed in research, clinical guidelines, and real-world practice. All drafts were subsequently examined and refined during a series of both virtual and face-to-face meetings. When discrepancies or divergent opinions arose, these were addressed through structured group discussion. If consensus could not be reached through dialogue alone, anonymous voting was conducted. The panel agreed in advance that consensus would be quantitatively defined as at least 80% agreement among the participants on each key recommendation. Once all subsections were finalized, the entire panel joined to ensure that the overall manuscript demonstrated consistency and clarity across all tumor types and age categories. The draft consensus document was then shared with external experts, who provided independent review and feedback, and this feedback was incorporated into the final version. Recognizing the biological and clinical heterogeneity of brain tumors throughout life, the manuscript is organized first by tumor type and subsequently stratified into four major age groups: pediatric (0–14 years); adolescent and young adult (15–39 years); adult (40–69 years); and elderly (70 years and older). Within each age group, the document reviews the most clinically relevant tumor entities and predictive biomarkers, explicitly commenting on their prognostic and therapeutic implications. The recommendations derive from synthesis of published evidence, alignment with internationally accepted guidelines, and consensus among experts in cases where evidence is limited or rapidly evolving. Additionally, the panel considered practical aspects of biomarker testing, including the availability and turnaround time for assays, and the importance of integrating results into multidisciplinary care pathways such as molecular tumor boards. This comprehensive, discursive approach provides a clear record of the consensus methodology and strengthens the scientific reliability of the document.

### Incidence and clinical utility of predictive biomarkers by age group

#### Pediatric patients (0–14 years)

The molecular landscape of pediatric brain tumors is distinct, with a higher prevalence of actionable genetic alterations compared to adults. In pediatric low-grade gliomas (pLGG), MAPK/ERK pathway alterations – most notably BRAF V600E mutations (found in up to 20–25% of pLGG) and KIAA1549–BRAF fusions (seen in 30–40%) – are the most frequent actionable events. These alterations predict response to both BRAF inhibitors (such as dabrafenib and vemurafenib) and MEK inhibitors (such as trametinib), which have received approval in the US and Europe for BRAF V600E-mutant pLGG [8, 13–15].

RAF inhibitors are a class of targeted therapies that specifically inhibit the activity of RAF kinases within the MAPK pathway, disrupting tumor growth signaling in tumors driven by BRAF mutations and fusions [16]More recently, the FDA has also granted accelerated approval for tovorafenib (Ojemda), the first systemic therapy for children with relapsed or refractory pLGG with either BRAF fusions/rearrangements or BRAF V600 mutations [17].

IDH mutations are rare in pediatric gliomas (< 5%), but when present, define a favorable subgroup with distinct biology and prognosis [18]. In infantile and congenital high-grade gliomas, gene fusions involving NTRK, ALK, ROS1, and FGFR are more prevalent (collectively up to 10–15% in this subgroup), and confer sensitivity to kinase inhibitors such as larotrectinib, entrectinib, and crizotinib [19].

Although NTRK fusions are rare in adult glioma patients (occurring in about 2% of cases, with a frequency ranging from 1.1 to2.6% in GBM) [20, 21], these alterations have gained notable attention in recent years due to the enhanced activity shown by specific inhibitors across different type of solid tumors. Larotrectinib is a highly selective inhibitor of TRK A, B, and C and was the first tumor-agnostic therapy approved by the European Medicines Agency (EMA). A pooled analysis described the results of two clinical trials enrolling pediatric and adult primary CNS tumor patients harboring NTRK gene fusions and treated with larotrectinib. Among the 33 enrolled patients, 26 were younger than 18 years. A recent international retrospective cohort analysis of patients with TRK fusion–driven CNS tumors confirmed the observed benefit from TRK inhibition is largely seen in pediatric patients, who had a median overall survival of 185.5 months compared with 24.8 months in adults [22, 23]. To date, several ongoing clinical trials and real-world registries are evaluating these agents in children with rare fusions (e.g., NCT02637687, NCT02576431). Diffuse midline gliomas in children are characterized by the K27M mutation, present in 70–80% of these cases, which serves as both a diagnostic hallmark and a poor prognostic indicator [24, 25]. While not directly targetable, this mutation is the focus of ongoing trials with agents such as ONC201 (dordaviprone)(NCT03295396). Notably, ONC201 received FDA accelerated approval on August 6, 2025, as the first and only therapy for recurrent H3 K27M-mutant diffuse midline glioma—including DIPG—in both pediatric and adult patients. This approval is based on efficacy data in patients at least one year old with progressive disease, providing a new and much-needed therapeutic option for this highly aggressive brain tumor type that previously had no approved treatment [26]. Another approach that has been widely used due to its effectiveness in oncology is immunotherapy, and in particular, the blockade of PD-1/PD-L1. Sporadic data showed the efficacy of anti-PD-1 inhibition in the rare setting of biallelic mismatch repair deficiency (bMMRD), a genetic predisposition leading to pediatric GBM with a very high mutational load. CDKN2A/B homozygous deletion, found in 5–10% of pediatric gliomas, is associated with high-grade transformation and poor prognosis [27]. Molecular subgrouping (WNT, SHH, Group 3, Group 4) is essential in medulloblastoma. The WNT subgroup (10–15% of cases) is associated with excellent prognosis and is a candidate for therapy de-escalation. SHH subgroup (25–30%) may benefit from SHH inhibitors, especially in PTCH1/SMO-mutated tumors, while Groups 3 and 4 (together 50–60%) lack approved targets but may harbor MYC/MYCN amplifications or PI3K/mTOR pathway activation, guiding trial inclusion [2, 28, 29]. Ependymomas in children are increasingly classified by molecular features. ZFTA–RELA fusions are present in 60–70% of supratentorial ependymomas, while YAP1–MAMLD1 fusions are less common but define a favorable prognosis. Posterior fossa ependymomas are stratified into PFA and PFB; PFA tumors (60–70% of pediatric cases) are characterized by EZHIP hypermethylation and H3K27me3 loss, associated with resistance to standard therapies and high recurrence risk [2, 30]. Pineoblastomas lack predictive biomarkers for treatment response but possess distinct prognostic molecular subgroups defined by alterations in DICER1/DROSHA, MYC/FOXR2, and RB1. Mutations in microRNA biogenesis genes (DICER1, DROSHA) are associated with more favorable outcomes, while MYC/FOXR2 amplifications or RB1 loss correlate with poor prognosis, especially in very young children. Genetic and epigenetic profiling is now fundamental for therapeutic and prognostic stratification in pineoblastoma [31].

Multiple international consortia—for example, INdividualized therapy FOr high-Risk childhood Malignancies (INFORM), Pediatric Neuro-Oncology Consortium (PNOC), and International Society of Paediatric Oncology (SIOP)—are actively enrolling children with rare molecular subtypes for targeted therapy evaluation.

#### Adolescent and young adult (AYA) patients (15–39 years)

AYA patients (15–39 years) present a unique molecular landscape that often overlaps with both pediatric and adult profiles. Pediatric-type gliomas, both high-grade and low-grade, can occur in this age group. IDH mutations become more frequent in AYA gliomas (up to 20–30%), with significant prognostic and therapeutic implications, including eligibility for IDH-targeted therapies and clinical trials [7].

BRAF alterations represents a relevant important in low-grade gliomas, with a prevalence similar to pediatric cases (15–20%) [32, 33]. The ROAR study, an open-label, single-arm, phase 2, basket trial demonstrated that the combination of dabrafenib and trametinib shows clinically meaningful activity and an acceptable safety profile in patients with BRAF V600E-mutant high-grade and low-grade gliomas [30, 31]. Importantly, these results specifically apply to adult patients, while the efficacy of dabrafenib plus trametinib in the pediatric population had already been established [34]. The ROAR study enrolled 45 patients older than 18 years with high-grade gliomas (HGGs) (69% GBM, 11% anaplastic pleomorphic xanthoastrocytoma, 11% anaplastic astrocytoma) and 13 with LGGs patients (31% ganglioglioma, 15% each of diffuse astrocytoma and pleomorphic xanthoastrocytoma). In the HGG cohort, after an independent, centralized radiology review, 14 patients (31%) achieved an objective response, including three complete responses (CR) and eleven partial responses (PR), with a median duration of response (DOR) of 13.6 months. Among GBM patients, Overall survival (OS) was 13.7 months. In the LGG cohort, nine patients (69%) had a response (1 CR, 6 PR, 2 minor responses), with a median DOR of 27.5 months. The median PFS was 14 months, while the median OS was not reached. As previously demonstrated in pediatric patients, tovorafenib has shown highly promising clinical efficacy in AYA patients with BRAF-altered low-grade gliomas. The FIREFLY-1 trial included patients aged 6 months to 25 years, and the consistency of positive outcomes across both pediatric and AYA populations highlights its potential as an effective treatment option in these groups [16].

NTRK rearrangements become increasingly rare with advancing age, and, in parallel, the clinical benefit of targeted therapies may also decrease. In the above mentioned pooled analysis in primary CNS tumor patients harboring NTRK gene fusions and treated with larotrectinib, only 7 patients were adults (≥ 18 years), with a 6-month DCR rate of 57% in HGG and 100% in LGG [21]; but no objective responses were seen in adult patients. Among the most representative CNS tumors in the AYA population, germ cell tumors (GCTs) display distinct molecular features with both prognostic and predictive significance.

Prognostic biomarkers include chromosome 12p gain, associated with unfavorable clinical outcomes, and DNA methylation profiles, which enable accurate distinction between germinomas and non-germinomatous GCTs [35]. From a predictive perspective, mutations in the KIT/RAS/MAPK pathway (particularly KIT and RAS) and alterations in the PI3K/AKT/mTOR pathway represent actionable molecular targets that may guide targeted therapy selection and support a personalized treatment approach. Furthermore, circulating microRNAs (notably miR-371a-3p) and immune checkpoint markers (PD-1 and PD-L1) are emerging as promising tools for disease diagnosis, treatment monitoring, and therapeutic stratification [36].

Of particular interest in the AYA population is the increased incidence of **mismatch repair (MMR) deficiency** and **hypermutator phenotypes**, estimated at 2–5% [37, 38]. These alterations are more frequent than in children or older adults and are especially relevant in the context of **hereditary cancer syndromes**, such as **Lynch syndrome (LS)** [39]. LS is a hereditary cancer predisposition syndrome caused by germline mutations in MMR genes (MLH1, MSH2, MSH6, PMS2, or EPCAM). While traditionally associated with colorectal and endometrial cancers, LS also predisposes individuals to primary brain tumors, particularly gliomas [40, 41]. Identifying LS in patients with brain tumors is essential for implementing targeted surveillance, enabling early detection in both patients and at-risk family members, who have a 50% chance of inheriting the mutation.

MMR-deficient tumors with microsatellite instability (MSI) often display high tumor mutational burden and may respond favorably to **immune checkpoint inhibitors** (e.g., anti-PD-1/PD-L1), making LS identification crucial in therapeutic planning. Diagnosis begins with immunohistochemical (IHC) staining to detect MMR protein loss, followed by germline testing to confirm LS and distinguish hereditary from sporadic cases [42]. Incorporating LS screening into the molecular evaluation of brain tumors supports personalized treatment approaches and enhances both prognostic accuracy and family risk assessment in the AYA population [43]. Another syndrome frequently encountered in this age group is Li-Fraumeni syndrome, due to germline mutations in TP53. LFS is associated with a broad spectrum of malignancies, including soft tissue sarcomas, breast cancer, adrenocortical carcinoma, and, in the neuro-oncological setting, an elevated risk of primary brain tumors such as astrocytomas and glioblastomas. Identifying LFS in patients with brain tumors is crucial to implementing individualized surveillance protocols and enabling early diagnosis both in affected individuals and their at-risk relatives [44].

In medulloblastoma, molecular subgroups remain clinically relevant across all ages, though their distribution shifts over time [45]. Among AYA patients, the WNT and SHH subgroups become less common, while Groups 3 and 4 are more frequently observed. Nevertheless, approximately 30% of medulloblastomas are still driven by abnormal activation of the **Sonic Hedgehog (SHH) signaling pathway**, making it a critical therapeutic target [31]. Currently, the most advanced treatment strategies targeting SHH signaling fall into two main categories: Smoothened (SMO) antagonists and GLI transcription factor inhibitors. SMO antagonists, such as vismodegib and sonidegib, are small molecules designed to block the SMO receptor, a key component of SHH signaling. These drugs have shown encouraging results, including tumor shrinkage and clinical improvement, both in preclinical studies and in some patients. Vismodegib, approved for basal cell carcinoma, has also shown efficacy in certain medulloblastoma cases, particularly in adults [46–48]. However, the clinical use of these agents is limited by several challenges. Resistance often develops rapidly, typically due to mutations in SMO itself or activation of downstream signaling components. Additionally, the blood-brain barrier poses a major obstacle, reducing drug penetration and limiting therapeutic efficacy in brain tumors. Side effects, particularly in pediatric patients, further complicate long-term treatment [49]. To address these limitations, researchers have turned their attention to GLI inhibitors, which target transcription factors downstream of SMO. These agents may bypass resistance mechanisms that undermine SMO antagonists. While promising, GLI inhibitors are still in early stages of development and face issues such as poor pharmacokinetics, limited brain bioavailability, and insufficient clinical data on their safety and efficacy in medulloblastoma patients [50]. As a result, ongoing research is focused on improving drug delivery systems to enhance the brain penetration and therapeutic impact of both SMO and GLI inhibitors. There is also growing interest in combination therapies that simultaneously target multiple points in the SHH pathway or incorporate other treatment modalities to overcome or prevent resistance [47]. Ultimately, the effective use of SHH pathway inhibitors depends on careful molecular profiling of individual tumors to identify patients who are most likely to benefit. Despite the existing challenges, SHH inhibitors represent a promising avenue for improving outcomes in this biologically distinct subgroup of medulloblastoma patients [40].

Basket trials and precision oncology programs are increasingly incorporating AYA patients who harbor rare or actionable alterations.

#### Adult patients (40–69 years)

In adults, the molecular landscape is dominated by biomarkers with established roles in diagnosis, prognosis, and therapy.

IDH1 and 2 mutations are the most clinically relevant predictive biomarkers in lower-grade gliomas, present in 70–80% of WHO grade 2/3 gliomas, and identifies a specific type of grade 4 glioma (astrocytoma grade 4 IDH mutant) [51]. The phase 3 INDIGO trial [11] demonstrated that vorasidenib, a IDH1 and IDH2 dual inhibitor, significantly prolonged progression-free survival (PFS) to 27.7 months compared to 11.1 months with placebo in patients with recurrent or progressive IDH-mutant grade 2 gliomas treated with a surgery alone approach, establishing a new practice-changing therapeutic option [11] Vorasidenib notably extended time to next intervention with a manageable safety profile. The INDIGO trial underscores the pivotal role of IDH-targeted therapies in altering the natural progression of IDH mutant low-grade gliomas. Other brain-penetrant IDH inhibitors, including those in early clinical development, are being investigated to expand treatment options and address resistance mechanisms. Incorporating vorasidenib and other emerging agents into treatment paradigms will refine the personalized management of IDH-mutant gliomas.

IDH mutations are associated with better prognosis and responsiveness to alkylating chemotherapy and emerging IDH inhibitors. However, risk criteria to define which patients are most likely to benefit with targeted agents remain under debate. MGMT promoter methylation is present in 35–45% of glioblastomas and serves as a key predictor of response to temozolomide [10, 52]. IDH-wildtype glioblastomas, which represent the majority of adult high-grade gliomas, frequently harbor EGFR amplification or mutation (40–50%), TERT promoter mutations (60–80%), and chromosome + 7/−10 copy number changes (70–80%). These alterations are important for tumor classification and prognostication, although effective targeted therapies are not yet available [53]. Homozygous deletion of CDKN2A/B is seen in 30–40% of adult glioblastomas and is associated with poor outcomes. RET, PTEN, and PI3K pathway alterations are observed in 10–20% of cases but currently lack effective targeted therapies [54]. BRAF V600 mutations can be also found in < 5% of gliomas. Adult patients were included in the ROAR trial, which demonstrated the potential efficacy of the dabrafenib-trametinib combination in this setting.

In meningiomas, NF2 mutations are common (40–60%) and associated with recurrence. Non-NF2 mutations—including TRAF7 (15–25%), KLF4 (10–15%), AKT1 (5–10%), SMO (5–10%), and PIK3CA (5–10%) – define distinct subgroups, some with potential for targeted therapy. PD-L1 expression is being explored as a biomarker for immunotherapy, especially in high-grade or refractory meningiomas [55].

Several basket, umbrella and platform trials are evaluating targeted therapies in adult patients with rare alterations. The ROAR study [56] is a basket trial focusing on rare brain tumors with the BRAF V600E mutation, including gliomas [30, 31]. Similarly, the REC-2282 trial [57] is a basket trial that targets NF2-mutated progressive meningiomas using the HDAC inhibitor AR-42. The N2M2/NOA-20 (NCT05214468) umbrella trial stratifies IDH-mutant gliomas based on molecular markers to tailor treatments. INSIGHT (NCT05058002) is an umbrella trial exploring targeted combination therapies in glioma subtypes. A071701 (NCT03001991) is an umbrella trial testing CDK4/6 inhibitors, such as abemaciclib, in recurrent high-grade gliomas that are stratified by molecular alterations.

VAGILE (NCT03970447), for example, is a global, adaptive platform trial for glioblastoma that evaluates multiple therapies in newly diagnosed and recurrent settings. It uses Bayesian adaptive randomization to optimize overall survival outcomes. These studies exemplify the integration of molecular profiling into trial design to optimize treatment efficacy and accelerate therapeutic discovery in neuro-oncology. Their diverse designs allow for the efficient stratification of patients and the evaluation of novel targeted agents across tumor subtypes.

Other molecular alterations that have been successfully targeted in different oncologic settings have also been investigated in brain tumors, but unfortunately without success. In particular c-Met and phosphatidylinositol 3-kinase (PI3K) signaling pathways are very frequently dysregulated in GBM. The loss of phosphatase and tensin homolog (PTEN), a negative regulator of PI3K, is a quite common alteration, occurring in around 25–40% of GBM [58]; MET overexpression can be found in about 30% of GBMs, while activating mutations and fusion genes were reported in 8–15% of HGGs [59]. However, in a Phase Ib/II study, the MET inhibitor INC280, as monotherapy or combined with buparlisib (PI3K inhibitor), showed no activity in PTEN-deficient/MET amplified recurrent GBM [1, 58].

Epidermal growth factor receptor (EGFR) overexpression or amplification, a molecular feature observed in about 30–60% of all GBM, and EGFR variant III (EGFR vIII), the most common EGFR activating mutation (25–64% of GBM) have been explored as potential therapeutic targets. In adults, results of trials investigating EGFR tyrosine kinase inhibitors and vaccination strategies against the EGFR vIII mutation have been disappointing so far [60, 61]. A recent phase 3 study evaluated the antibody–drug conjugate depatuxizumab mafodotin in newly diagnosed GBM patients with centrally confirmed EGFR amplification [55, 56], but no overall survival benefit was observed with the experimental treatment.

Among other solid cancers in adults, MMRd/MSI and tumor mutational burden (TMB) are well-established predictive biomarkers of response to immunotherapy, leading to multiple FDA and EMA agnostic approvals in both first-line and recurrent settings. However, in the pivotal phase 2, multi-cohort KEYNOTE 158 study, 13 enrolled patients with recurrent MMRd/MSI brain tumors showed no objective response to pembrolizumab, with a median PFS of 1.1 months and an OS of 5.6 months [38]. Therefore, apart from hereditary cancer syndromes, such as LS, TMB/MMR deficiency testing should be offered only when clinical trials are available [9].

#### Elderly patients (*≥* 70 years)

MGMT promoter methylation retains predictive value for temozolomide response and is present in 30–40% of elderly glioblastomas. TERT promoter mutations and EGFR amplifications are more frequent in this group (up to 80% and 50%, respectively) [62, 63]. Adding temozolomide (TMZ) to hypofractionated radiotherapy improves overall survival in glioblastoma patients aged 60 years and older, though the benefit is more evident in patients with MGMT promoter-methylated tumors. Combined chemoradiation remains the preferred option, but TMZ monotherapy may be considered as a second-line approach for MGMT-methylated patients unable to tolerate or not eligible for combined treatment [5]. Recent data show only minor gains in overall survival for MGMT-unmethylated tumors with combined therapy, and the negative CheckMate 498 trial highlights ongoing uncertainties regarding optimal management for this population.

Clinical trials of targeted therapies in the elderly remain limited due to comorbidities and underrepresentation; however, real-world data and registry-based studies are beginning to address this gap (Table 1).

**Table 1:** Summary of key actionable biomarkers identified in brain tumors across pediatric, adolescent/young adult, and adult age groups

Age Group	Tumor Type	Key Actionable Biomarkers (Prevalence)	Clinical Utility / Trials
Pediatric	pLGG	BRAF V600E (20–25%), KIAA1549–BRAF (30–40%)	Targeted therapy (BRAF/MEK inhibitors)
	pHGG	NTRK/ALK/ROS1/FGFR fusions (10–15%)	Kinase inhibitors; ongoing trials
	Diffuse midline	K27M (70–80%)	Diagnostic, prognosis, ONC201 trials
	Medulloblastoma	WNT (10–15%), SHH (25–30%), MYC/PI3K	Risk stratification, SHH inhibitors
	Ependymoma	ZFTA–RELA (60–70%), YAP1–MAMLD1, PFA (60–70%)	Subgrouping, prognosis, trials
AYA	Glioma	IDH (20–30%), BRAF (15–20%), MMR (2–5%)	Prognosis, targeted therapy, immunotherapy
	Medulloblastoma	Subgroup markers	Risk stratification
Adult	Lower-grade glioma	IDH (70–80%), 1p/19q (60–70%)	Diagnosis, prognosis, trials
	Glioblastoma or astrocytoma IDH mutant grade 4	IDH (10%), MGMT (35–45%), EGFR amplification (40–50%), TERT (60–80%) mutation, CDKN2A/B hom deletion (30–40%)	Prognosis, therapy response
	Meningioma	NF2 (40–60%), TRAF7, KLF4, AKT1, SMO, PIK3CA	Subgrouping, targeted therapy trials
Elderly	Glioblastoma	IDH (< 10%), MGMT (30–40%), TERT (up to 80%), EGFR (up to 50%)	Prognosis, therapy response

Note: Prevalence data are approximate and may vary by cohort and testing methods. For rare subtypes, consult ongoing clinical trials and registry studies for current therapeutic opportunities

### Sequencing platforms

The EANO guidelines for rational molecular testing in targeted therapy selection recommend considering local resources, clinical trial opportunities, patient status, and disease stage as central factors when designing molecular testing strategies [9] as these conditions ultimately impact access to targeted treatments. Decision-making should also account for the type and expected frequency of genetic alterations: frequent hotspot mutations are best investigated with target-specific assays, whereas broader sequencing techniques are advisable when multiple, less common genetic alterations may be present [9].Recent EANO updates [1]highlight the increasing clinical application of Next Generation Sequencing (NGS), driven by technological improvements that have cut costs and turnaround times. NGS enables concurrent detection of mutations, fusions, copy number changes, an*d* molecular signatures, thus gathering extensive molecular data without requiring multiple single-target assays, which often consume more tissue, time, and resources [1, 9]. Numerous NGS panels are now available worldwide. While many are pan-cancer and few have been specifically designed for brain tumors, several contain key genes (such as BRAF, IDH1/2, FGFR1-3, NTRK1-3) and signatures (tumor mutational burden) of direct relevance for neuro-oncology [9]. The selection of the appropriate panel should consider input material, technology, comprehensiveness, and clinical setting. US-based companies and international providers (e.g., Foundation One^®^ CDx, MSK-IMPACT^®^, Guardant360 Tissue Next™) offer centralized analysis services, which are suitable options for sites with limited resources, enabling access to comprehensive profiling for single cases. Larger hospitals can often run NGS in-house, offering more rapid and cost-effective molecular diagnostics to greater patient numbers. Most tissue-based NGS assays operate on DNA from FFPE sections, delivering reliable results with minimal input (as little as 10 ng DNA, e.g., Oncomine™ Dx Target Test), allowing comprehensive molecular profiling and informed therapeutic targeting even on small biopsies (Table 2). Main commercial panels cover hundreds of genes (e.g., Foundation One^®^ CDx, MSK-IMPACT^®^, TruSight™ Oncology Comprehensive) and can report multigene signatures (e.g., TMB, MMR, HRD) in a single test. Narrower panels offering very short turnaround times (e.g., Oncomine™ Dx Express Test, Myriapod^®^ NGS Cancer panel DNA) facilitate timely clinical decision-making. While NGS from FFPE tissue remains the standard for profiling, liquid biopsy panels based on circulating cell-free DNA (ccfDNA) are emerging as complementary tools, particularly for patients ineligible for surgery or those requiring molecular reassessment at recurrence. However, plasma-based liquid biopsies for brain tumors often yield low levels of tumor-derived DNA due to the blood-brain barrier, making comprehensive detection challenging. Recent studies demonstrate that CSF is a far richer source of circulating tumor DNA (ctDNA) for neuro-oncology liquid biopsies. The yield and molecular fidelity of CSF ctDNA outstrip those of plasma, as CSF is generally more enriched with tumor-derived DNA, especially when collected near the tumor or from intraventricular locations. Sensitivities above 90% have been reported for detection of key mutations and molecular subtypes using NGS or digital PCR in CSF. CSF-based liquid biopsy facilitates noninvasive diagnosis, longitudinal molecular monitoring, tracking of tumor evolution, and prognostication in patients with primary and metastatic brain tumors. These advances are particularly significant for scenarios with inaccessible tissue or non-contributory plasma assays [64]. Alongside mainstream NGS approaches, nanopore sequencing technologies are rapidly advancing in neuro-oncology. Nanopore platforms enable real-time sequencing and analysis, providing rapid tumor classification and DNA methylation profiling within minutes to hours, even from very small biopsy samples. The long-read capabilities of nanopore sequencing allow direct mapping of complex genetic and epigenetic events, comprehensive profiling of novel fusions, methylation patterns, and copy-number variations. These benefits are driving interest for intraoperative or urgent diagnostics, and nanopore workflows are now being validated globally for brain tumor subtyping, despite regulatory standards still being under debate. As costs and operational barriers fall, nanopore sequencing is expected to further transform the precision medicine landscape in brain tumors-even for liquid biopsy applications from CSF.​By incorporating CSF liquid biopsy and nanopore sequencing into the molecular diagnostic algorithm, clinicians gain powerful new options for comprehensive, minimally invasive genomic profiling and dynamic molecular monitoring. This facilitates adaptive therapeutic strategies and expands access to precision neuro-oncology, regardless of current regulatory boundaries.

Advances in molecular testing now allow detailed profiling from minimal tumor material, yet these insights have not translated into broadly available or effective targeted therapies. Most remain investigational and accessible only via clinical trials [1, 9].

**Table 2:** Main NGS platforms approved by regulatory agencies (FDA, CE-IVD) for clinical use for pan-cancer profiling from tumor tissue or plasma

Biological source	NGS panel	Setting	Biological material	Technology	Genetic signatures			N° of genes	Genes covered by the panel relevant for therapeutic targeting in brain tumors	Reference
					TMB	HRD	MMRD			
TUMOR TISSUE	Foundation One^®^ CDx	Centralized assay	DNA from FFPE tumor tissue	Illumina	Yes	Yes	Yes	324	ALK, BRAF, CDK4/6, CTNNB1, EGFR, FGFR1-3, H3F3A, IDH1/2, MDM2/4, MET, NF1, NTRK1-3, PDGFRA, PI3KCA, RET, ROS1, SMO, SUFU, TSC1/2	https://www.rochefoundationmedicine.com/#/en/international/foundationone-cdx
	MSK-IMPACT^®^	Centralized assay	DNA from FFPE tumor tissue	Illumina	No	Yes	Yes	468	ALK, BRAF, CDK4/6, CTNNB1, EGFR, FGFR1-3, H3F3A, HIST1H3B, IDH1/2, MDM2/4, MET, NF1, NTRK1-3, PDGFRA, PI3KCA, RET, ROS1, SMO, SUFU, TSC1/2	https://www.accessdata.fda.gov/cdrh_docs/reviews/den170058.pdf
	Tempus xT Cdx Assay by Tempus Lab Inc.	Centralized assay	DNA from FFPE tumor tissue (and matched gDNA)	Illumina	No	Yes	Yes	648	ALK, BRAF, CDK4/6, CTNNB1, EGFR, FGFR1-3, H3F3A, HIST1H3B, IDH1/2, MDM2/4, MET, NF1, NTRK1-3, PDGFRA, PI3KCA, RET, ROS1, SMO, SUFU, TSC1/2	https://www.tempus.com/life-sciences/xt-cdx/?srsltid=AfmBOopuZeT7HNM735jmmwiVVJKKKhGBUybPE67jmIj7YxK06JM_1NRQ
	Guardant360 Tissue Next™	Centralized assay	DNA from FFPE tumor tissue	-	Yes	Yes	Yes	498	ALK, BRAF, CDK4/6, CTNNB1, EGFR, FGFR1-3, H3F3A, IDH1/2, MDM2/4, MET, NF1, NTRK1-3, PDGFRA, PI3KCA, RET, ROS1, SMO, SUFU, TSC1/2	https://www.guardantcomplete.com/products/guardant360-tissuenext
	TruSight™ Oncology Comprehensive	In-house sequencing	DNA and RNA from FFPE tumor tissue	Illumina	Yes	Yes	Yes	517	ALK, BRAF, CDK4/6, CTNNB1, EGFR, FGFR1-3, H3F3A, HIST1H3B, IDH1/2, MDM2/4, MET, NF1, NTRK1-3, PDGFRA, PI3KCA, RET, ROS1, SMO, SUFU, TSC1/2	https://emea.illumina.com/products/by-type/ivd-products/trusight-oncology-comprehensive.html
	Oncomine™ Dx Target Test	In-house sequencing	DNA from FFPE tumor tissue	Ion Torrent, Thermo Fisher Scientific	No	No	No	46	ALK, BRAF, CDK4, CTNNB1, EGFR, FGFR1-3, IDH1/2, KRAS, MET, NTRK1-3, PDGFRA, PIK3CA, RET, ROS1, SMO	https://www.thermofisher.com/it/en/home/clinical/diagnostic-testing/condition-disease-diagnostics/oncology-diagnostics/oncomine-dx-target-test/oncomine-dx-target-test-us-only.html
	OncoDEEP^®^ by OncoDNA	In-house sequencing	DNA from FFPE tumor tissue	Illumina, MGI, Elements Bioscience	Yes	Yes	Yes	638	ALK, BRAF, CDK4/6, CTNNB1, EGFR, FGFR1-3, H3F3A, HIST1H3B, IDH1/2, MDM2/4, MET, NF1, NTRK1-3, PDGFRA, PI3KCA, RET, ROS1, SMO, SUFU, TSC1/2	https://oncodna.com/for-laboratories/solid-biopsy-oncodeep/
	OncoScreen™ Plus by Burning Rock Dx	In-house sequencing	DNA from FFPE tumor tissue	Illumina	Yes	Yes	Yes	520	ALK, BRAF, CDK4/6, CTNNB1, EGFR, FGFR1-3, H3F3A, HIST1H3B, IDH1/2, MDM2/4, MET, NF1, NTRK1-3, PDGFRA, PIK3CA, RET, ROS1, SMO, SUFU, TSC1/2	https://us.brbiotech.com/p_details.php?class_id=102101101
	Myriapod^®^ NGS Cancer panel RNA	In-house sequencing	RNA from FFPE tumor tissue	Illumina	No	No	No	10	ALK, FGFR2-3, MET ex14 skipping, NTRK1-3, RET, ROS1	https://www.diatechpharmacogenetics.com/en/myriapod-ngs-line-dry/
TUMOR TISSUE/ LIQUID BIOPSY	Myriapod^®^ NGS Cancer panel DNA	In-house sequencing	DNA from FFPE tumor tissue or ccfDNA	Illumina	No	No	No	17	ALK, BRAF, EGFR, FGFR3, IDH1/2, MET, PDGFRA, PIK3CA, RET, ROS1	https://www.diatechpharmacogenetics.com/en/myriapod-ngs-line-dry/
	Oncomine™ Dx Express Test	In-house sequencing	DNA from FFPE tumor tissue or ccfDNA	Ion Torrent, Thermo Fisher Scientific	No	No	No	46	ALK, BRAF, CDK4, CTNNB1, EGFR, FGFR1-3, IDH1/2, MET, NTRK1-3, PDGFRA, PIK3CA, RET, ROS1	https://www.thermofisher.com/it/en/home/clinical/diagnostic-testing/condition-disease-diagnostics/oncology-diagnostics/oncomine-dx-express-test.html
LIQUID BIOPSY	Foundation One^®^ Liquid CDx	Centralized assay	ccfDNA	Illumina	Yes	Yes	Yes	324	ALK, BRAF, CDK4/6, CTNNB1, EGFR, FGFR1-3, H3F3A, IDH1/2, MDM2/4, MET, NF1, NTRK1-3, PDGFRA, PIK3CA, RET, ROS1, SMO, SUFU, TSC1/2	https://www.rochefoundationmedicine.com/#/en/international/foundationone-liquid-cdx
	Guardant360^®^ CDx	Centralized assay	ccfDNA	Illumina	No	No	No	74	ALK, BRAF, CDK4/6, CTNNB1, EGFR, FGFR1-3, IDH1/2, MET, NF1, NTRK1/3, PDGFRA, PI3KCA, RET, ROS1, SMO, TSC1	https://www.guardantcomplete.com/assets/pdf/Guardant360-CDx-Technical-Information-US.pdf
	Oncocompass™ Target by Burning Rock Dx	In-house sequencing	ccfDNA	Illumina	No	Yes	Yes	168	ALK, BRAF, CDK4/6, CTNNB1, EGFR, FGFR1-3, HIST1H3B, IDH1/2, MET, NF1, NTRK1-3, PDGFRA, PIK3CA, RET, ROS1	https://us.brbiotech.com/p_details.php?class_id=102101103

Abbreviations: ccfDNA = circulating cell-free DNA; CE-IVD = Conformité Européenne - In Vitro Diagnostic; FDA = Food and Drug Association; FFPE = formalin-fixed paraffin-embedded; gDNA = genomic DNA; HRD = homologous recombination deficiency; MMRD = mismatch repair deficiency; NGS = Next Generation Sequencing; TMB = tumor mutational burden

## Discussion

**Fig. 2:**
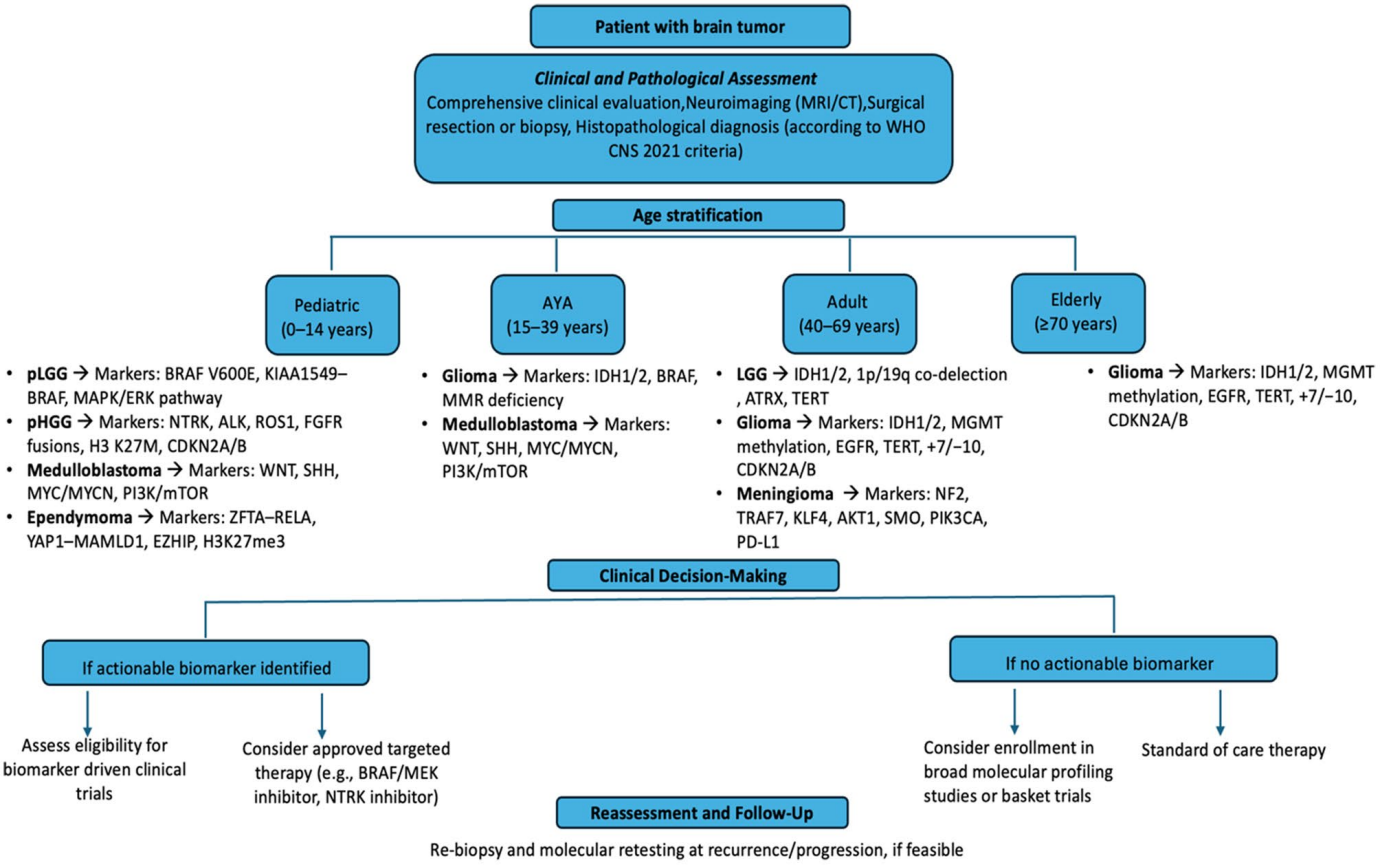
Decision-making algorithm for the management of brain tumors based on age group and presence of actionable biomarkers

The spectrum of predictive molecular biomarkers in brain tumors is profoundly influenced by patient age, reflecting the underlying biological diversity of CNS neoplasms across the lifespan (Fig. 2). Our consensus confirms that pediatric tumors are enriched for gene fusions and MAPK pathway alterations, which not only define distinct molecular subgroups but also represent actionable targets for therapies such as BRAF and MEK inhibitors. In contrast, adult and elderly patients more commonly harbor IDH, TERT, and EGFR mutations, with established roles in diagnosis, prognosis, and, in some cases, therapy selection. The AYA population bridges these biological extremes, displaying overlapping molecular features from both pediatric and adult profiles.

Despite these advances, several challenges limit the full implementation and clinical impact of precision medicine in neuro-oncology. One major barrier is the **limited access to comprehensive molecular diagnostics in low-resource settings**. The availability of next-generation sequencing, methylation profiling, and advanced immunohistochemistry is often restricted by cost, infrastructure, and the need for specialized personnel. This disparity can result in delayed or suboptimal diagnosis, missed opportunities for targeted therapy, and inequitable patient outcomes. Addressing these gaps will require investment in laboratory infrastructure, workforce training, and the development of cost-effective, scalable testing platforms.

Another challenge is the **low prevalence of actionable biomarkers in adult and elderly populations**, with less than 5% of patients eligible for currently approved targeted therapies. This underscores the need for ongoing translational research to identify new molecular targets and develop effective agents for these subgroups. Additionally, the interpretation ofVUS remains a significant hurdle, often requiring functional validation and expert consensus to determine clinical relevance.

**Emerging technologies** offer potential solutions to several of these limitations. Liquid biopsy approaches, including circulating tumor DNA (ctDNA) and RNA analysis, are being explored for non-invasive molecular profiling, disease monitoring, and detection of resistance mutations.

DNA methylation profiling has profoundly transformed the classification of central nervous system (CNS) tumors, providing diagnostic refinement and improved risk stratification, even in challenging or ambiguous cases. Multiple technologies–such as whole-genome bisulfite sequencing, microarrays (Illumina EPIC), enzymatic methyl-sequencing, and third-generation sequencing like Oxford Nanopore–have demonstrated high accuracy in defining molecular CNS tumor subtypes, often impacting clinical decision-making and enhancing diagnostic confidence.​ This methodology is particularly valuable for cases with limited or poor-quality tissue, and for rare or pediatric tumors where histological diagnosis may be uncertain and the risk of diagnostic discordance is significant. By integrating methylation profiling with traditional molecular and histopathological approaches, clinicians can detect epigenetic alterations associated with specific WHO grades, enabling early identification of malignant transformation and supporting individually tailored therapeutic strategies.​.

As DNA methylation profiling becomes more accessible and widely validated–including automated classifiers and real-time analysis–its clinical applications are expanding from diagnosis and risk stratification to dynamic tumor monitoring, assessment of therapeutic response, and the identification of emerging molecular biomarkers. These advances promise to further enhance precision medicine initiatives and facilitate real-time, adaptive management of CNS tumors [35].

The **importance of international data sharing and collaboration** cannot be overstated. Large, harmonized registries and collaborative research networks –such as INFORM, PNOC, and SIOP in pediatrics, and the EANO and cIMPACT-NOW consortia in adults –are essential for aggregating data on rare molecular subtypes, validating novel biomarkers, and accelerating the translation of discoveries into clinical benefit. These efforts should be complemented by open-access data platforms, standardized reporting, and the inclusion of diverse patient populations to ensure generalizability and equity.

Finally, the central role of the **Molecular Tumor Board (MTB)** is reaffirmed by our consensus. MTBs facilitate the integration of complex molecular data with clinical context, prioritize actionable alterations, and guide individualized therapy decisions –including clinical trial enrollment for patients with rare or investigational biomarkers. Expanding access to MTBs, especially through virtual platforms, may help bridge gaps in expertise and resource availability across institutions and regions. In summary, while the integration of predictive biomarkers has already improved the classification, risk stratification, and –in select cases –treatment of brain tumors, the full promise of precision neuro-oncology will only be realized through continued research, innovation, and multidisciplinary collaboration. Addressing disparities in access, embracing emerging technologies, and fostering international cooperation are critical steps toward optimizing outcomes for patients with brain tumors at every stage of life.

### Recommendations

Based on the current evidence, international guidelines [1, 2] and expert consensus, we propose the following numbered recommendations to inform clinical practice:

1. **Implement Routine Age-Adapted Molecular Testing**:
  Comprehensive molecular profiling should be performed for all patients with brain tumors, with testing strategies tailored to age and tumor type. If not previously tested for diagnostic purposes, priority should be given to biomarkers with established clinical utility (e.g., IDH mutations, MGMT promoter methylation, BRAF alterations, NTRK fusions), as recommended by the WHO CNS 2021 and EANO guidelines.
2. **Leverage Multidisciplinary Molecular Tumor Boards**:
  The interpretation of complex molecular findings and the selection of targeted therapies should be guided by multidisciplinary MTBs, which integrate expertise from neuro-oncology, pathology, molecular genetics, and other specialties. MTBs are particularly critical for rare, complex, or uncertain alterations (EANO, 2025).
3. **Encourage Clinical Trial Enrollment**:
  Enrollment in clinical trials should be actively promoted for patients with rare or investigational biomarkers, especially in pediatric and AYA populations, where actionable targets are more prevalent and standard options may be limited. Participation in trials provides access to novel therapies and contributes to the advancement of evidence-based care.
4. **Harmonize Testing Protocols and Reporting Standards**:
  Institutions should strive to harmonize molecular testing protocols, reporting standards, and interpretation criteria across age groups. Standardization, as outlined in international guidelines, enhances result reliability, enables multicenter collaboration, and ensures equitable access to high-quality diagnostics.
5. **Support Ongoing Research and Innovation**:
  Continued investment in translational and clinical research is essential to expand the therapeutic landscape, especially for adult and elderly patients, who currently have fewer actionable targets and are often underrepresented in trials.

### Limitations

While this consensus statement is comprehensive and grounded in current evidence, several important limitations must be acknowledged. One major concern is the underrepresentation of elderly patients and minority populations in molecular studies and clinical trials. This gap may limit the generalizability of the recommendations and raise issues regarding equity in the application of precision medicine advances. Additionally, the field of molecular neuro-oncology is evolving at a rapid pace, with new biomarkers and therapeutic options continuously emerging. As a result, some of the current recommendations may need to be revised or updated as new evidence becomes available. Another challenge relates to disparities in resources and access; advanced molecular diagnostic techniques and targeted therapies are not uniformly available across all regions and institutions, which could hinder the universal implementation of these guidelines. Furthermore, interpreting the clinical significance of many rare or novel biomarkers, including VUS, remains complex and may vary between experts and centers. These limitations underscore the ongoing need for research, regular updates to clinical guidelines, and concerted efforts to improve inclusivity and equitable access within the field of neuro-oncology.

### Future directions

Several key priorities will define the future direction of precision neuro-oncology. First and foremost, it is essential to expand equitable access to comprehensive molecular profiling, ensuring that all patients, regardless of their age or geographic location, can benefit from high-quality molecular testing. Alongside this, accelerating the develoPMIent of targeted therapies remains a critical goal, particularly focusing on novel agents that address currently untreatable molecular alterations, with special attention to adult and elderly populations who have historically been underrepresented in research. Another important direction involves promoting age-specific clinical trials that better reflect the biological diversity of brain tumors across the lifespan. Complementing these trials, the use of real-world evidence should be leveraged to inform and refine clinical practice. To improve clinical decision-making, advancing the interpretation of VUS is necessary; this can be achieved by developing consensus frameworks and functional assays that clarify their relevance. International collaboration and data sharing are also vital to accelerate biomarker discovery and validation. Supporting the creation of large, harmonized registries and collaborative research networks will enhance the pace and quality of research in this field. Finally, engaging patients and caregivers as active partners in research prioritization, clinical trial design, and guideline development is crucial to ensure that scientific advances align closely with patient needs and values. Together, these priorities will help drive the evolution of precision neuro-oncology toward more inclusive, effective, and patient-centered care.

## Conclusions

Age is a key determinant of the molecular landscape in brain tumors, shaping both the incidence and clinical utility of predictive biomarkers. Precision medicine approaches must be tailored to the biological and clinical context of each age group to optimize outcomes and equity. Multidisciplinary collaboration, ongoing research, and a commitment to inclusivity are essential to realize the promise of precision neuro-oncology for all patients. By embracing innovation, harmonizing practices, and centering patient voices, the neuro-oncology community can advance personalized care and improve outcomes across the lifespan.

## Data Availability

No datasets were generated or analysed during the current study.
